# Programmed Death Ligand 1 (PD-L1) and Tumor-Associated Macrophages in Gastric-Type Hepatocellular Carcinoma: Prognostic Insights

**DOI:** 10.3390/ijms27136048

**Published:** 2026-07-06

**Authors:** Rita Szodorai, Ilona Kovalszky, Katalin Dezső, Simona Gurzu

**Affiliations:** 1 Department of Pathology, George Emil Palade University of Medicine, Pharmacy, Science and Technology, 540139 Targu Mures, Romania; rita.szodorai@umfst.ro; 2 Department of Pathology, Clinical County Emergency Hospital, 540136 Targu Mures, Romania; 3 Department of Pathology and Experimental Cancer Research, Semmelweis University, 26. Ulloi ut, H-1085 Budapest, Hungary; kovalszky.ilona@med.semmelweis-univ.hu (I.K.); dezso.katalin@semmelweis.hu (K.D.); 4 Research Center of Oncopathology and Translational Research (CCOMT), 540136 Targu Mures, Romania; 5 Romanian Academy of Medical Sciences, 030167 Bucuresti, Romania

**Keywords:** hepatocellular carcinoma, PD-L1, epithelial-mesenchymal transition, tumor-associated macrophages

## Abstract

Hepatocellular carcinoma (HCC) is a heterogeneous primary liver malignancy characterized by limited treatment options and low overall survival rates. Recent studies have explored the role of programmed death ligand 1 (PD-L1), tumor-associated macrophages (TAMs), and epithelial-mesenchymal transition (EMT) in modulating tumor progression and the response to immunotherapy. This study aimed to investigate the association among PD-L1 expression, TAMs, and EMT in HCC, highlighting the recently proposed immunophenotypic variant—gastric-type HCCs. A retrospective cohort of 50 surgically resected HCC patients was analyzed. Immunohistochemical staining was performed for PD-L1 (clones 28-8 and 22C3), CD68 (TAMs), and EMT markers (VSIG-1, TTF-1, and vimentin). PD-L1 expression was detected in 52% of the patients and was significantly associated with high TAM counts (*p* < 0.001). Compared with PD-L1-negative patients, those with gastric-type HCCs, which are characterized by VSIG-1 and TTF-1 co-expression and vimentin negativity, demonstrated improved survival outcomes (*p* = 0.03). Integration of immune and EMT profiling of tumor cells in routine diagnostics may guide prognosis and immunotherapeutic strategies in HCC. Further molecular validation is required to confirm the biological significance of the proposed gastric-type HCC immunophenotype.

## 1. Introduction

Hepatocellular carcinoma (HCC) remains a major therapeutic challenge despite significant advancements in diagnostic techniques and therapeutic modalities [1]. Its molecular and clinical heterogeneity results in suboptimal patient outcomes [2]. The 5-year survival rate is only 18% [1,2,3]. Most patients present at advanced stages, often with cirrhosis, which limits the feasibility of curative treatment options. In addition to multiple available therapeutic strategies, the current management of HCC focuses on immunotherapy, particularly immune checkpoint inhibitors (ICIs) [4,5,6]. However, long-term outcomes remain poor, and regional recurrences and distant metastases are frequent. Recently, clinical trials have highlighted the significant oncological benefits of inhibitors targeting programmed cell death ligand 1 (PD-L1) in various malignancies [5,6]. As several clones of PD-L1 are available, it is not yet defined which clone is most appropriate for predicting response to immunotherapy in HCC.

The low response rates of HCC patients to ICIs also involve intrinsic or acquired resistance mechanisms. The presence of tumor-associated macrophages (TAMs) in the tumor microenvironment (TME) is supposed to play a key role in promoting tumor progression and modulating therapeutic resistance [7].

Another mechanism contributing to HCC progression is epithelial–mesenchymal transition (EMT). This process induces phenotypic changes in epithelial cells, characterized by the loss of polarity and intracellular adhesion and the acquisition of mesenchymal features that increase migratory and invasive behaviors. EMT is immunohistochemically characterized by overexpression of mesenchymal markers such as N-cadherin, vimentin (VIM), and Snail, and by downregulation of epithelial markers such as E-cadherin, V-set and immunoglobulin domain-containing 1 (VSIG-1), and TTF-1 [8,9,10].

This study aimed to investigate the prognostic significance of PD-L1 expression on immune cells (ICs) and tumor cells (TCs) in HCC using two available commercial clones. The immunohistochemical expression of PD-L1 was examined across the histological subtypes of HCC according to the World Health Organization (WHO) classification; however, as original and preliminary data, we also assessed its positivity in one of the newly described histological forms, known as gastric-type HCCs. They are characterized by cytoplasmic expression of VSIG and TTF-1 and VIM negativity and were hypothesized to be associated with longer overall survival [9,10]. Additionally, we propose exploring the relation between TAMs and PD-L1 expression as a possible mechanism of tumor progression and therapeutic resistance [2,5].

## 2. Results

### 2.1. Clinicopathological Parameters

The study cohort included 50 patients diagnosed with HCC. A male predominance was observed (37 males vs. 13 females). The age of the patients ranged from 9 to 91 years, with a median age of 65.82 years. Unifocal tumors were observed in 68% of the patients (n = 34). The underlying lesions were found to be frequently associated. Cirrhosis was found in over half of the cases (n = 28); 10 of these patients presented with chronic hepatitis, and, in the other 18, alcoholic cirrhosis was diagnosed.

**Figure 1:**
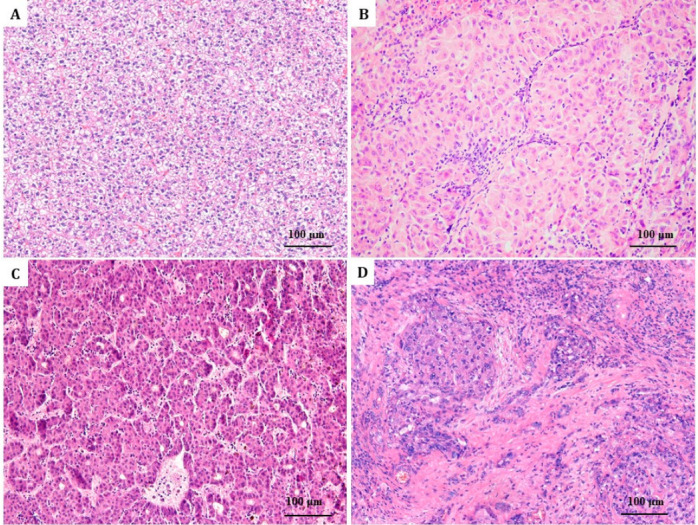
Histological subtypes of HCC. (**A**) Clear-cell subtype (H&E, 20×); (**B**) trabecular (H&E, 20×); (**C**) acinar (pseudoglandular) (H&E, 20×); (**D**) scirrhous (H&E, 20×).

Four histological subtypes of HCC were identified based on the 5th edition of the WHO Classification of Tumors of the Digestive System [11]: clear-cell type (42%; n = 21), defined by optically clear cytoplasm with centrally located round nuclei and minimal stromal content; trabecular (38%; n = 19), characterized by thick hepatic cords separated by sinusoidal-like vascular spaces, mimicking normal liver architecture; acinar (14%; n = 7), exhibiting gland-like structures with luminal spaces, surrounded by a delicate stroma; and scirrhous (6%; n = 3), which shows an abundant fibrous stroma with scattered nests or cords of tumor cells (Figure 1).

**Figure 2:**
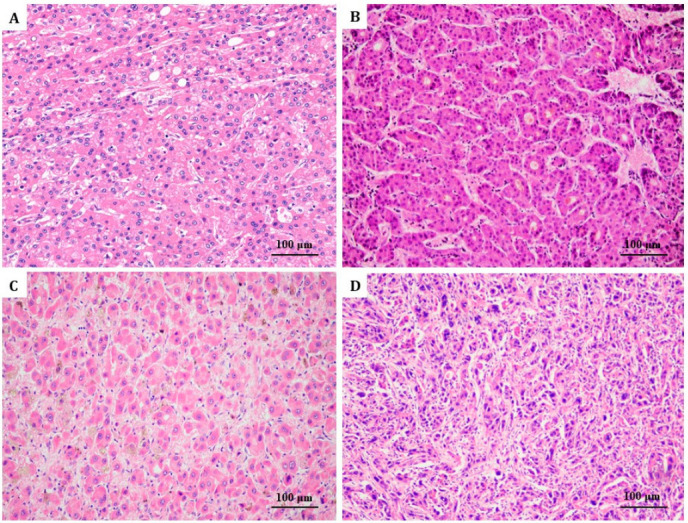
Histologic grading system used for HCC. (**A**) Grade 1 (well differentiated) (H&E, 20×); (**B**) Grade 2 (moderately differentiated) (H&E, 20×); (**C**) Grade 3 (poorly differentiated) (H&E, 20×); (**D**) Grade 4 (undifferentiated) (H&E, 20×).

Only 2% of the cases were well differentiated; 42% were moderately differentiated; 46% showed poor differentiation of tumor cells; and 10% were undifferentiated (Figure 2). Vascular invasion was detected in 18/50 patients (36%).

Based on the 8th edition of the AJCC tumor staging system [11], a similar distribution was seen between the three stages: stage I (52%; n = 26), stage II (34%; n = 17), and stage III (14%; n = 7). Patients diagnosed with stage IV were excluded from the study. The immunohistochemical analysis revealed VSIG-1 positivity in 37 cases (74%) and TTF-1 expression in 39 of the 50 HCCs (78%). Vimentin expression was seen in six cases.

### 2.2. PD-L1 Expression and TAMs

**Figure 3:**
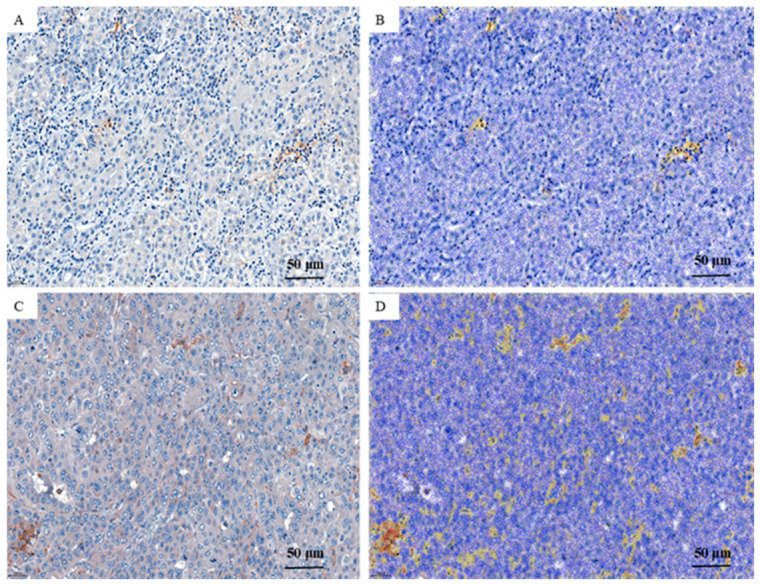
PD-L1 expression in lymphocytes was evaluated with an optical microscope (**A**), magnification 40× and quantified with QuantCenter software version 3.0 (**B**); PD-L1 expression in tumor cells was also evaluated with an optical microscope (**C**), magnification 40× and quantified with the software (**D**).

In our cohort of 50 HCC cases, 26 were PD-L1 positive (Figure 3). Of them, 12 cases (24%) expressed clone 28-8 only (Table 1) and 12 cases (24%) were positive for both clones (28-8 and 22C3) (Table 2). Two cases (4%) were positive only for clone 22C3. The remaining 24 cases (48%) were double negative for both clones.

Comparative analyses were performed between these groups across clinicopathologic and immunohistochemical parameters. No statistically significant differences were identified between PD-L1-positive and -negative cases concerning patient age, gender, tumor size, histologic subtype, tumor grade, pT stage, vascular invasion, multicentricity, or underlying liver disease (steatosis, cirrhosis, or hepatitis) (*p* > 0.05).

Of the 12 cases showing PD-L1 positivity with clone 28-8, PD-L1 expression was found in tumor cells in seven cases (58.3%) and in peritumoral lymphocytes in five cases (41.7%). Among the 12 double-positive cases, PD-L1 was expressed in tumor cells in eight cases (66.7%) and in peritumoral lymphocytes in four cases (33.3%).

A statistically significant association was observed between PD-L1 28-8 positivity and CD68+ macrophage density. Tumors with PD-L1 28-8 expression had a higher median percentage of CD68+ cells (9.8%) than PD-L1-negative tumors (3.0%; *p* = 0.0018). This finding suggests that PD-L1 expression by tumor cells correlates with an immune microenvironment enriched in macrophages.

When comparing double-positive PD-L1 cases (both clones 28-8 and 22C3 positive, n = 12) with double-negative tumors (n = 24), a more pronounced difference was observed. Double-positive tumors showed significantly higher CD68+ macrophage density (median 28.3% vs. 2.9%, *p* = 0.00012). In contrast, no other clinicopathological or survival parameter, including cirrhosis and vascular invasion, was significantly associated with double PD-L1 positivity (*p* > 0.05).

Only two cases represented the 22C3-only positive group. Due to the small sample size, no robust statistical conclusions could be drawn for this subset, and the results should be interpreted with caution.

**Figure 4:**
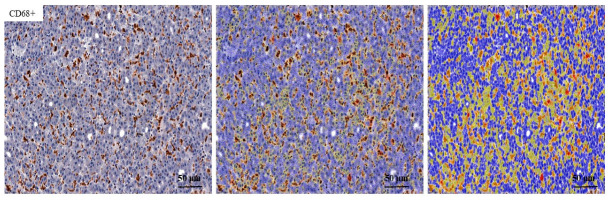
In PD-L1-positive HCCs, TAM-rich cases are characterized by the presence of CD68-positive macrophages within ≥15% of the area analyzed. The CD68-positive macrophages can be seen using an optical microscope (left—brown cells) but are quantified with QuantCenter software—middle and right (caption: ob. × 40).

**Figure 5:**
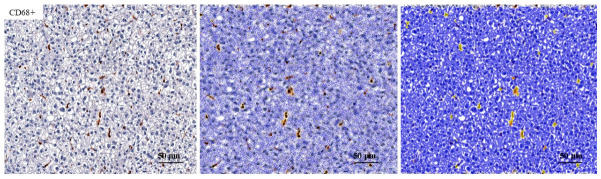
CD68-positive macrophages (<15%) in PD-L1-negative cases (caption: ob. × 40), evaluated with optical microscope (**left**) and quantified with the QuantCenter (**middle** and **right**). The yellow–brownish areas represent positive staining of the macrophages.

**Figure 6:**
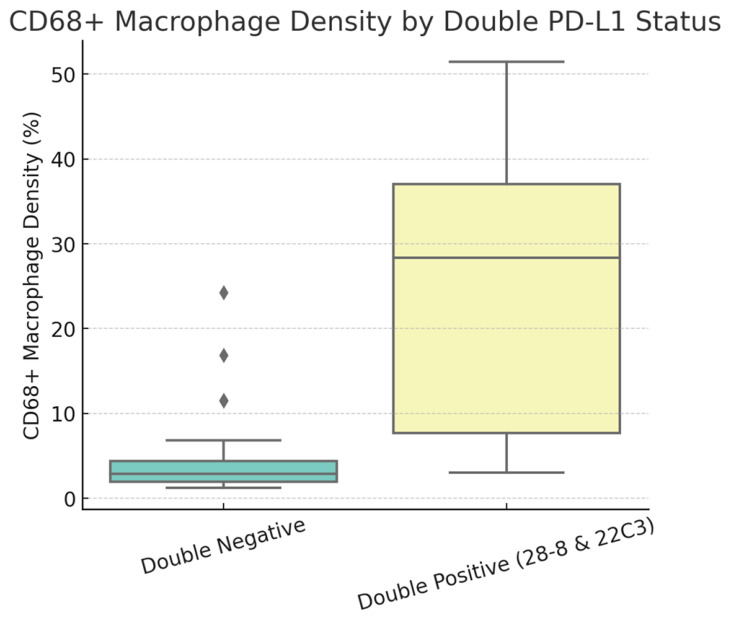
Box plot analysis of CD68+ macrophage density. Double-positive HCCs (positivity for PD-L1—clones 28-8 and 22C3, n = 12) show a significantly higher CD68+ macrophage infiltration (median 28.3%) compared with double-negative (n = 24) tumors (median 2.9%)—Mann–Whitney U test, *p* = 0.00012.

The range of CD68+ macrophages in PD-L1-positive cases was between 15% and 52% (Figure 4), whereas the proportion of CD68+ cells ranged between 1.79% and 14.84% in PD-L1-negative HCCs (Figure 5). A direct correlation was seen between PD-L1 positivity and TAMs (Figure 6).

These findings suggest that PD-L1 expression is closely associated with a higher degree of macrophage infiltration within the tumor. However, it does not show a significant correlation with other clinicopathological features, such as cirrhosis, vascular invasion, or tumor stage.

### 2.3. Survival Rate

**Figure 7:**
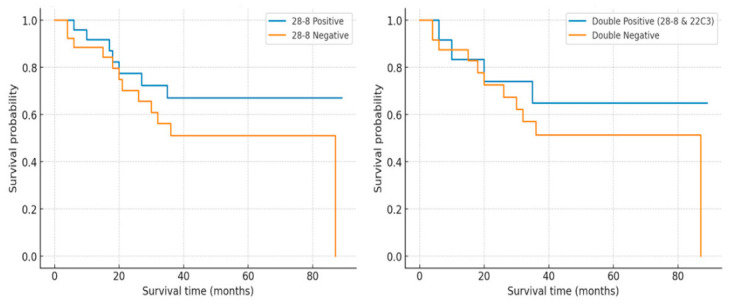
(**Left**) Kaplan–Meier survival curves show no statistical differences between PD-L1-positive versus PD-L1-negative HCCs (*p* = 0.22). (**Right**) Although the survival rate of the double-positive group tends to be longer compared with the double-negative group, the difference does not reach statistical significance (*p* = 0.37).

The Kaplan–Meier survival curves demonstrated improved overall survival in PD-L1-positive patients (median 47.5 months), with a longer median survival than in PD-L1-negative patients (median 31 months). However, this difference did not reach statistical significance (*p* = 0.22). While PD-L1 expression was associated with features of an immune-enriched tumor microenvironment, it was not identified as an independent predictor of overall survival in the present study (Figure 7).

### 2.4. Gastric-Type HCCs: Clinicopathological Features, PD-L1 Expression, and TAMs

We investigated the clinicopathological features of gastric-type HCCs, a novel histological subtype defined by double positivity for TTF-1 and VSIG-1 in the cytoplasm and negativity for VIM [9,10]. We analyzed PD-L1-positive cases (28-8 and 22C3), PD-L1-negative cases, and the density of tumor-associated macrophages (TAMs) within the tumor.

Out of the 50 analyzed cases, 33 were classified as gastric-type HCCs. Among these, 18 cases (54.5%) were negative for PD-L1 expression and 15 cases (45.5%) were positive. The positive cohort included nine cases (27.3%) demonstrating 28-8 positivity and six cases (18.2%) that were double positive (28-8 and 22C3). As the WHO does not yet recognize this new immunophenotypic subtype of HCC, these preliminary results should be confirmed in a larger cohort of patients (Table 3).

The overall analysis showed no significant differences in age, gender, tumor size, or survival among the three groups. Tumor grade differentiation, additional lesions, vascular invasion, tumor architecture, staging, and histological type were not significantly associated with PD-L1 status. Notably, CD68+ macrophage infiltration demonstrated significant variation between groups (*p* < 0.001), with the highest levels observed in double-positive PD-L1 tumors.

When PD-L1-negative and PD-L1-double-positive tumors were compared, significant differences were observed in age (*p* = 0.032), tumor size (*p* = 0.032), and survival (*p* = 0.032). No significant associations were found for gender, tumor differentiation, vascular invasion, or tumor architecture (*p* ≈ 0.28). When comparing 28-8-positive and -negative cases, only age differed significantly (*p* = 0.039), whereas tumor size (*p* = 0.67), survival (*p* = 0.44), and clinicopathological parameters (all *p* > 0.4) did not differ significantly.

The quantitative evaluation revealed significant differences in CD68+ macrophage density across the three groups (*p* = 0.024). The pairwise comparisons revealed a borderline difference between negative and double-positive tumors (*p* = 0.076), whereas comparisons of 28-8 positive vs. negative (*p* = 0.45) and 28-8 positive vs. double positive (*p* = 0.22) were not statistically significant.

These results suggest that double PD-L1 positivity is associated with younger age and improved survival compared to PD-L1-negative cases, as well as with enriched CD68+ macrophage infiltration compared to PD-L1-negative tumors. In contrast, positivity for 28-8 alone was associated only with younger age. These findings highlight the potential prognostic impact of dual PD-L1 expression and its association with the immune microenvironment in gastric-type HCCs.

**Figure 8:**
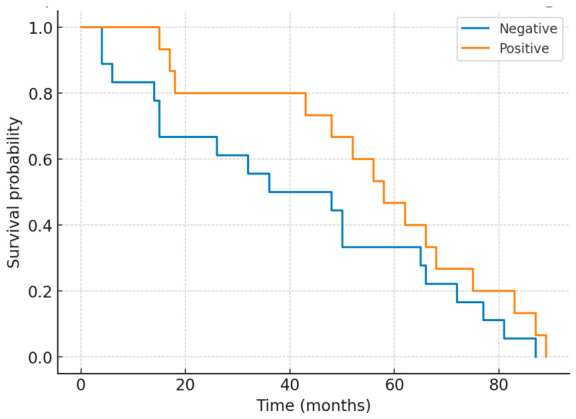
In the Kaplan–Meier survival analysis of gastric-type HCCs, patients with PD-L1-positive tumors demonstrated improved survival compared with PD-L1-negative cases (*p* = 0.02).

The Kaplan–Meier survival analysis supported these findings, showing that patients with PD-L1-positive tumors had better survival rates compared to those with PD-L1-negative tumors (Figure 8). The survival curve for PD-L1-positive cases shifted to the right, indicating longer median and mean survival times: 58 months versus 42 months and 55.8 months versus 41.6 months. In our cohort, PD-L1-positive gastric-type HCCs showed a favorable survival trend; however, given the limited sample size, these findings should be considered preliminary. The Cox regression analysis showed that PD-L1 expression was not an independent predictor of overall survival in the overall cohort. In the univariate analysis, pT stage (HR = 2.14, *p* = 0.014), high tumor grade (HR = 3.59, *p* = 0.024), vascular invasion (HR = 3.13, *p* = 0.017), and the gastric-type immunophenotype (HR = 0.21, *p* = 0.002) were significantly associated with overall survival. In the reduced multivariate Cox model including PD-L1 28-8 expression, pT stage, tumor grade, and vascular invasion, only high tumor grade remained independently associated with poorer survival (HR = 4.67, *p* = 0.009), whereas PD-L1 expression did not retain independent prognostic significance. Within the gastric-type HCCs, PD-L1 positivity indicated a favorable survival trend (HR = 0.13); however, this association did not reach conventional statistical significance (*p* = 0.056). Therefore, the observed survival differences should be considered exploratory and require validation in larger cohorts.

## 3. Discussion

Several immune checkpoint blockade therapies have been used in clinical trials, with a focus on anti-PD-1 and anti-PD-L1 regimens. Pharmacological inhibition of the PD-1/PD-L1 axis increases T-cell activation and cytotoxicity, thereby significantly impeding the progression of several malignancies, including HCC [6,12].

PD-L1 seems to be linked with the EMT phenomenon across various malignant tumors, including lung carcinomas, melanomas, colorectal adenocarcinomas, esophageal squamous cell carcinomas, extrahepatic cholangiocarcinomas, head and neck tumors, breast cancers, renal cell cancers, and glioblastomas [13,14,15,16]. However, the relationship between PD-L1 expression and EMT remains incompletely understood. The efficacy of PD-L1 inhibitors is inconsistent, potentially due to the bidirectional relationship between PD-L1 expression and EMT status [5].

Jung et al. demonstrated a correlation between PD-L1 expression in tumor cells (PD-L1 TCs) and aggressive disease phenotypes, such as deep tumor invasion and microvascular involvement. However, PD-L1 TC expression does not affect overall survival. In contrast, PD-L1 expression in immune cells (PD-L1 IC) is positively associated with improved survival in HCC patients, although the underlying mechanism remains incompletely understood. Furthermore, their study highlighted a significant association between EMT status and tumor recurrence rate. PD-L1 IC staining intensity was lower in EMT-negative cases than in EMT-positive cases, suggesting that EMT plays a crucial role in the early steps of metastasis [5]. As observed in other tumors, the acquisition of mesenchymal phenotypes enhances the invasive capacity of cancer cells, facilitating stromal infiltration and dissemination to distant organs, ultimately contributing to metastasis and recurrence [5,17].

The evaluation of PD-L1 expression has become clinically significant, primarily due to its role in selecting patients for ICI therapy. However, accurate assessment remains challenging due to intratumoral heterogeneity. IHC analysis is performed using various PD-L1 clones, including 22C3, 28-8, SP142, and SP263 [18,19]. Positivity for clone SP142 is an indicator of response to atezolizumab, whereas SP263 is associated with durvalumab treatment [20]. Clone 22C3 was approved by the FDA for pembrolizumab therapy. Although clone 28-8 has also been clinically validated for anti-PD-L1 therapy, the data regarding this clone are generally rare.

Consistent with our data, PD-L1 expression in HCC was more frequently detected with clones 28-8 and 22C3 than with other clones, such as SP142 and SP263, suggesting clone-dependent variability [18,21].

In our study, clone 28-8 and the double positive cases were prioritized due to their more consistent staining, particularly in immune cells within the tumor microenvironment. As PD-L1 clone 28-8 is a marker of the extracellular domain, which is expressed by immune cells rather than HCC cells, it can be hypothesized that this clone may provide a higher detection rate of PD-L1 expression; however, its predictive value for immunotherapy response remains to be established. Similarly, Kashif et al., using both the 22C3 and 28-8 clones, concluded that testing with clone 28-8 may be beneficial when 22C3 yields negative results, emphasizing the importance of using complementary clones in PD-L1 evaluation [19].

We also revealed a positive correlation between CD68+ TAM density and PD-L1 expression in both tumor cells and immune cells. This suggests a potential link between the two parameters of the HCC microenvironment. This finding reflects an immunologically active tumor phenotype but requires validation in larger cohorts. Based on these trends, we hypothesize that macrophage-rich tumors may be associated with PD-L1 upregulation and potentially influence the response to immune checkpoint blockade.

Shigeta et al. also emphasized the role of TAMs and EMT in the context of PD-1 therapy [18]. They reported that TAMs upregulate N-cadherin and vimentin expression in HCC cells while downregulating the epithelial marker E-cadherin, thereby promoting metastatic progression. CD68+ TAMs can promote EMT through paracrine signaling, including the secretion of TGF-β and IL-10, as well as through direct cell interactions [7,18]. Subsequently, EMT activation can upregulate PD-L1 via the ZEB1, STAT3, and β-catenin pathways, thereby modifying immune tolerance [22]. These studies have suggested that TAM-rich mesenchymal-like HCCs may be associated with a more immunosuppressive phenotype and therapeutic resistance. Our results also describe associations among CD68+ TAMs, PD-L1 expression, and vascular invasion.

Moreover, anti-PD-1 therapy not only reduces immunosuppression but also induces M1 macrophage polarization, enhancing antitumor effects. In addition to the PD-1/PD-L1 axis, CD47 has emerged as a macrophage-associated checkpoint and a marker of poor prognosis in patients with HCC. The interaction between CD47 and signal regulatory protein α (SIRPα) enables tumor cells to evade macrophage-mediated phagocytosis. CD47 blockade can reverse this evasion, restoring macrophage-mediated tumor suppression. These findings underscore the importance of macrophage-related checkpoints in HCC and highlight the need for further investigation into mechanisms that contribute to disease progression [7,18].

In our previous study, we examined EMT in HCC using TTF-1, VSIG-1, and VIM [10]. We previously described gastric-type HCCs as a putative immunophenotypic subgroup characterized by co-expression of TTF-1 and VSIG-1 and a lack of vimentin expression. Although this newly described entity is not yet recognized in the WHO classification of liver tumors, our findings, along with those of previous studies, suggest a pattern that requires further molecular profiling and validation. The patients exhibiting this immunophenotypic profile tended to have more favorable survival outcomes than those with the triple-negative profile. Compared with PD-L1-negative HCCs, PD-L1-positive HCCs were associated with a higher density of CD68+ macrophages. A trend toward improved survival was observed, although this did not reach statistical significance in the overall cohort. Further molecular or transcriptomic profiling is needed to determine whether the observed immunophenotypic patterns reflect biologically meaningful subgroups of HCC.

The limitations of the study include the small sample size, particularly within the novel described subgroup of gastric-type HCCs and dual PD-L1-positive tumors. Furthermore, the conclusions are derived predominantly from morphological, immunohistochemical, and clinicopathological observations, without integrated molecular validation. Therefore, the proposed biological significance of gastric-type HCCs and the prognostic importance of PD-L1 expression should be regarded as hypothesis-generating observations that need further confirmation in a larger cohort with molecular characterization. Another limitation is that macrophage profiling was restricted to CD68 immunostaining, which provides only a general assessment of macrophage infiltration. Although CD68 is widely used as a macrophage marker, it does not distinguish between different macrophage polarization states. Therefore, future studies should incorporate methods to separately detect M1 and M2 macrophage populations using markers such as CD163 and CD206 to better characterize the functional macrophage landscape in HCC.

The prognostic associations observed in the present study, including the survival trends identified in PD-L1-positive gastric-type HCCs, should be interpreted with caution because of the limited sample size. The Cox regression analysis further supports the exploratory nature of the survival findings. Although favorable survival trends were observed in selected PD-L1-positive subgroups, PD-L1 expression was no longer an independent prognostic factor after adjustment for established clinicopathological variables. Therefore, the prognostic significance of PD-L1 expression in HCC requires validation in larger cohorts.

To ensure reliable assessment of PD-L1 expression, further investigations should incorporate standardized scoring systems and validated concordance studies across different antibody clones.

## 4. Materials and Methods

### 4.1. Patient Selection

In this study, we evaluated the clinicopathological characteristics of patients diagnosed with HCC between 2016 and 2023. The study was approved by the Ethics Committee of the Emergency Clinical County Hospital of Târgu Mureș, Romania, and the Ethics Committee of Semmelweis University, Hungary. The inclusion criterion was a minimum follow-up duration of 4 months. Patients who received oncologic treatment prior to surgical intervention were excluded.

Individual patient profiles were created by compiling relevant clinical and pathological characteristics, including age, gender, tumor classification, histologic grade, and tumor stage [11]. Histopathological evaluation for all patients was performed according to the criteria outlined in the 5th edition of the World Health Organization (WHO) Classification of Tumors of the Digestive System.

### 4.2. Immunohistochemical Evaluation and Interpretation

Immunohistochemical (IHC) staining was performed on formalin-fixed paraffin-embedded (FFPE) liver tissue samples containing both HCC tumor cells and adjacent nontumoral hepatic parenchyma. Standardized IHC protocols were applied to FFPE sections via an automated staining system. A panel of markers, including VSIG-1, TTF-1, VIM, PD-L1 (clones 22C3 and 28-8), and CD68, was selected to assess epithelial, mesenchymal, and immune-related features.

The staining intensity and distribution were independently evaluated by two pathologists using brightfield microscopy. In cases of discrepant interpretation, the slides were reassessed together, and a consensus score was assigned before inclusion in the statistical analyses. Quantification was based on predefined thresholds, including a ≥5% cutoff for cytoplasmic expression of epithelial and mesenchymal markers, ≥1% positivity for PD-L1 expression in tumor and immune cells, and ≥15% positivity for CD68-positive tumor-associated macrophages. For PD-L1 evaluation, a positivity threshold of ≥1% was selected based on previously published studies in HCC and commonly applied immunotherapy-related assessment criteria. Because no universally accepted PD-L1 scoring system has been established for HCC, several approaches, including the tumor proportion score (TPS), combined positive score (CPS), and immune-cell-based assessment, have been reported in the literature. Given the retrospective design and the relatively small cohort size, PD-L1 expression was evaluated using a simplified binary classification (positive versus negative) to facilitate consistent comparisons across cases.

Internal positive controls were used to ensure the validity of the staining. Additionally, to complete manual scoring, representative slides were subjected to digital image analysis using a high-resolution whole-slide scanning system with dedicated quantitative pathology software [10]. In PD-L1-negative cases, a supplemental block was used, and the immunohistochemical stain was repeated. This approach enabled the objective validation of marker expression.

### 4.3. Statistical Analysis

Statistical analysis was performed using GraphPad Prism software (version 8). The correlations between clinicopathological characteristics and IHC markers were established using Pearson χ^2^, chi-square, and Fisher’s exact tests. Statistical significance was defined as *p* < 0.05. Overall survival (OS) rates were estimated via the same software, complemented by Kaplan–Meier curves. The median follow-up period after surgery was 42 months.

## 5. Conclusions

This manuscript emphasizes the correlation between PD-L1 expression, TAMs and EMT-related markers in HCC, with particular attention to a recently proposed gastric-type immunophenotypic pattern. PD-L1 expression proved to be associated with increased infiltration of CD68-positive macrophages, suggesting a potential interaction between the two parameters of the HCC microenvironment.

The observed associations were particularly notable in cases showing gastric-type HCCs, characterized by VSIG-1 and TTF-1 positivity and vimentin negativity. However, given the limited cohort size, these findings should be considered preliminary. At present, gastric-type HCCs should be considered as a proposed immunophenotypic category that requires further biological characterization.

Although differences in survival were observed in selected subgroup analyses, the prognostic significance of PD-L1 expression and gastric-type HCCs remains uncertain and warrants confirmation in larger independent cohorts. Similarly, while clone 28-8 demonstrated a higher rate of PD-L1 detection in our series, additional studies are needed to determine its potential value in comparison with other validated PD-L1 assays.

## Figures and Tables

**Table 1:** The clinicopathological features of HCC in PD-L1-positive (clone 28-8) versus PD-L1-negative cases.

Parameters	28-8 Positive (n = 12)	PD-L1 Negative (n = 24)	*p*-Value
**Age (** **year** **), median (range)**	65.5 (54–75)	68.0 (39–76)	0.45
**Gender (M/F)**	11/1	18/6	1.0
**Tumor architecture** (Unifocal/Multifocal)	7/5	14/10	0.47
**pT stage** (pT1/pT2/pT3)	7/3/2	11/9/4	0.94
**Histological type** (Trabecular/Acinar/Clear/Scirrhous)	5/1/5/1	9/2/12/1	0.42
**Grade** (G1 + G2/G3 + G4)	5/7	12/12	0.54
**Vascular invasion** (Present/Absent)	3/9	10/14	0.71
**Liver cirrhosis** (Present/Absent)	8/4	10/14	0.08
**Hepatitis B or C** (Yes/No)	3/9	6/18	0.83
**Vimentin** (Negative/Positive)	11/1	23/1	0.58
**TTF-1** (Negative/Positive)	1/11	5/19	0.59
**VSIG-1** (Negative/Positive)	3/9	5/19	0.80
**CD68+ macrophages median** (%)	4.1 (15–52)	2.9 (1–14.84)	0.001

**Table 2:** The clinicopathological features of HCC in double PD-L1-positive (clones 28-8 and 22C3) versus PD-L1-negative cases.

Parameters	Double Positive (n = 12)	PD-L1 Negative (n = 24)	*p*-Value
**Age (** **year** **), median (range)**	66.0 (9–81)	68.0 (39–76)	0.98
**Gender (M/F)**	7/5	18/6	0.29
**Tumor architecture** (Unifocal/Multifocal)	10/2	14/10	0.34
**pT stage** (pT1/pT2/pT3)	6/5/1	11/9/4	0.72
**Histological type** (Trabecular/Acinar/Clear/Scirrhous)	4/4/3/1	9/2/12/1	0.13
**Grade** (G1 + G2/G3 + G4)	4/8	12/12	0.60
**Vascular invasion** (Present/Absent)	5/7	10/14	1.0
**Liver cirrhosis** (Present/Absent)	10/2	10/14	0.23
**Hepatitis B or C** (Yes/No)	1/11	6/18	0.45
**Vimentin** (Neg/Pos)	9/3	23/1	0.28
**TTF-1** (Neg/Pos)	3/9	5/19	1.0
**VSIG-1** (Neg/Pos)	4/8	5/19	0.30
**CD68+ macrophages** median (%)	28.4 (18–52)	2.9 (1–14.84)	0.0001

**Table 3:** Clinicopathological features of gastric-type HCCs in PD-L1-positive vs. -negative cases.

Parameters for Gastric-Type HCCs	28-8 Clone (n = 9)	Double Positive (n = 6)	Negative (n = 18)	*p* Value
Age (year), median (range)	67.5	65.94 (9–81)	65.87 (58–76)	0.25
**Gender:** male/female	8/1	2/4	12/6	0.08
**Tumor architecture:** unifocal/multifocal	5/4	6	12/6	0.17
**Tumor size (mm, mean ± SD)**	36.12	39.63	42.76	0.61
**pT stage—8th AJCC edition** (1/2/3)	5/2/2	5/1	7/11	0.38
**Histological type** trabecular/acinar/clear cell	4/1/4	3/1/2	7/2/9	0.51
**Grade of differentiation:** low/high	5/4	3/3	10/8	0.97
**Vascular invasion:** present/absent	3/6	1/5	4/14	0.72
**Liver cirrhosis:** present/absent	6/3	5/1	7/11	0.11
**CD68+ macrophages**	1.30–52.09%	>15% (15.21–45.07%)	<15% (1.79–14.84%)	<0.001

## Data Availability

The original contributions presented in this study are included in the article. Further inquiries can be directed to the corresponding author.
